# Endoscopic Revision (StomaphyX) versus Formal Surgical Revision (Gastric Bypass) for Failed Vertical Band Gastroplasty

**DOI:** 10.1155/2013/108507

**Published:** 2013-01-22

**Authors:** Johan Bolton, Richdeep S. Gill, Akram Al-Jahdali, Simon Byrns, Xinzhe Shi, Daniel W. Birch, Shahzeer Karmali

**Affiliations:** ^1^Department of Surgery, University of Alberta, Edmonton, AB, Canada T6G 2B7; ^2^Department of Surgery, University of British Columbia, Vancouver, British Columbia, Canada; ^3^Faculty of Medicine and Dentistry, University of Alberta, Edmonton, AB, Canada T6G 2B7; ^4^Center for the Advancement of Minimally Invasive Surgery (CAMIS), Community Services Center, Royal Alexandra Hospital, Room 405, 10240 Kingsway, Edmonton, AB, Canada T5H 3V9

## Abstract

*Background*. Weight regain secondary to VBG pouch dilation is a typical referral for Bariatric surgeons. In this study we compare an endoluminal pouch reduction (Stomaphyx) to RYGB for revision. *Methods*. A retrospective review was completed for patients with a previous VBG presenting with weight regain between 2003–2010. *Results*. Thirty patients were identified for study 23 RYGB, 14 StomaphyX. Significant post procedure BMI loss was seen in each cohort (RYGB, 47.7 ± 7 kg/m^2^ to 35 ± 7 kg/m^2^; StomaphyX 43 ± 10 kg/m^2^ to 40 ± 9 kg/m^2^, *P* = 0.0007). Whereas nausea and headache were the only complications observed in StomaphyX patients, the RYGB group had a 43.5% complication rate and 1 mortality. Complications following RYGB include: incisional hernia (13%), anastomotic leak (8.7%), respiratory failure (8.7%), fistula (8.7%), and perforation (4.35%). The median length of stay following RYGB was 6 days compared to 1.5 ± 0.5 days following StomaphyX. *Conclusion*. This study suggests that while RYGB revision may achieve greater weight loss, the complication rates and severity is discouraging. StomaphyX may be a safe alternative. Further technical modifications of the device and longer follow-up may clarify the role of this approach.

## 1. Introduction

According to the World Health Organization, there are over 500 million obese individuals worldwide [1]. Bariatric surgery has been shown to be an effective treatment strategy to produce marked weight loss in patients with moderate to severe obesity (BMI > 35 kg/m^2^) [2]. Bariatric surgery includes both primarily restrictive and malabsorptive procedures. Specifically, vertical banded gastroplasty (VBG), a primarily restrictive bariatric surgical procedure, was first described by Mason in 1982 [3]. Despite initial optimism with VGB, long-term results have been disappointing. According to Balsiger et al., only 20% of patients maintained 50% excess weight loss (EWL) at 10-year followup [4]. Weight regain following VBG may be related to staple-line dehiscence and stomal pouch dilation [5]. In addition, reoperation and revision of VBG are needed in 20% to 30% of patients [4, 6]. 

Roux-en-Y gastric bypass (RYGB) remains the most common revisional procedure following failed VGB. RYGB had been shown to produce marked weight loss as a revisional procedure for previously failed restrictive bariatric surgical procedures [7–9]. However, some studies have suggested increased complication rates with revisional bariatric operations as high as 12% to 41% ([10] Schwartz RW, Obes Surg 2002; [11]. Recently, an endoscopic treatment strategy involving the StomaphyX device (Endogastric Solutions Inc., Redmond, WA, USA) has been used to revise gastric pouch dilatations. Endoscopic revision via the StomaphyX has been reported in patients following VBG and RYGB successfully [12, 13]. Our objective was to retrospectively compare weight loss and complication rates, following revision of failed VGB with either the StomaphyX device or formal surgical revision to RYGB at our institution. 

## 2. Materials and Methods

A retrospective review was completed for all patients with a previous VBG presenting to a comprehensive adult weight management clinic (Weight Wise) with weight regain between 2003 and 2010. The multidisciplinary team at Weight Wise including physicians, nurses, physiotherapy, and dieticians assessed these patients. Patients with previous VGB that presented with persistent weight gain despite conservative measures and meeting the Canadian Guidelines for Surgical Intervention were considered for revision by StomaphyX or open conversion to RYGB [14]. VBG revision endoscopically via the Stomaphyx device was performed as a part of a clinical trial, with the results previously reported by Manouchehri et al. [12]. 

### 2.1. StomaphyX Revision of VBG

StomaphyX is an endoluminal device that has recently been developed as an alternative to revisional surgery. A gastroscope is inserted through the internal lumen of the device and full-thickness gastric tissue is suctioned into the device allowing for the application of a polypropylene fastener which if repeated circumferentially creates a circular pleat thus downsizing the gastric pouch. The StomaphyX device has since been used and studied at our institution as a minimally invasive revisional option for patients with failed VBG [12]. 

### 2.2. Preoperative Characteristics

Patient demographics were collected retrospectively, including age, sex, and mean preoperative weight and body mass index (BMI) following the initial VBG. 

### 2.3. Outcomes

The primary outcomes of interest were complication rates after revisional procedure weight loss. This included perioperative and postoperative complications such as anastomotic leakage, intra-abdominal abscess, dehiscence, respiratory or cardiac complications, and incisional hernia. Mortality was also recorded. Weight loss following bariatric revisional surgery was recorded. Also, the operative time to complete either VBG revision with StomaphyX or formal conversion to RYGB was recorded, along with length of hospital stay (LOS). 

### 2.4. Statistical Analysis

Descriptive statistics were reported as mean ± standard deviation. Comparison of pre- and postbariatric procedure outcomes was performed using a paired Student *t*-test. Statistical significance was defined as *P* < 0.05. 

## 3. Results

**Figure 1:**
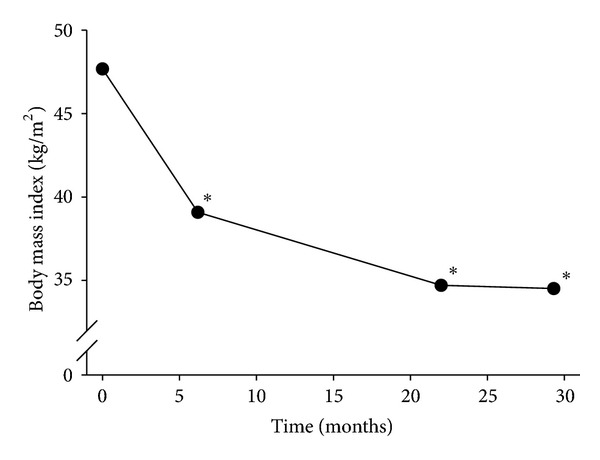
Change in body mass index (BMI) following formal conversion of VBG to RYGB. *P < 0.05 versus preoperative body mass index.

**Figure 2:**
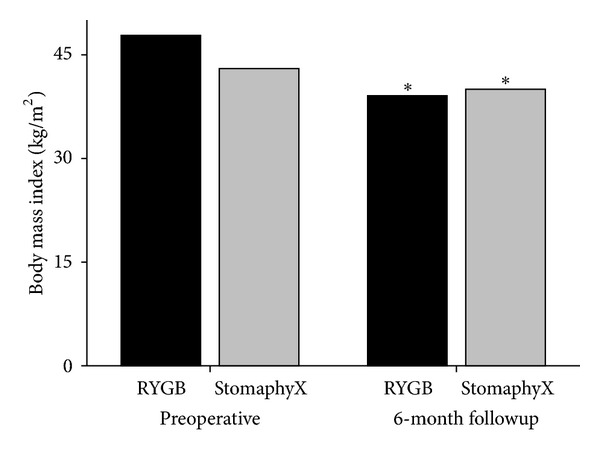
Comparison of initial body mass index and at 6-month followup in VBG patients revised by RYBG or endoscopically (StomaphyX). *P < 0.05 versus preoperative body mass index.

There were a total of thirty-seven patients from 2003 to 2010 that were included in the analysis. The preoperative characteristics are detailed in Table 1. Twenty-three patients were identified that had previously failed VBG and conversion to RYGB. Fourteen patients were identified with previously failed VBG and endoscopic revision via StomaphyX. The preoperative BMI in the RYGB group was 47.7 ± 7 kg/m^2^ compared to 43 ± 10 kg/m^2^ in the StomaphyX group. As seen in Figure 1, patients with previously failed VBG had a significant decrease in BMI following RYGB (47.7 ± 7* *kg/m^2^ to 35 ± 7* *kg/m^2^ at 24-month followup, *P* = 0.0007). In patients following StomaphyX endoscopic revision, there was also a significant decrease in BMI (43 ± 10 kg/m^2^ to 40 ± 9 kg/m^2^ at 6-month followup, *P* = 0.0007).  Figure 2 demonstrates the changes in BMI in both groups at 6-months followup. 

The only complications observed in the StomaphyX group were short-term nausea and headache (Table 2). On the other hand, the RYGB group had an overall 43.5% complication rate with a postoperative mortality. Major complications in the RYGB group include anastomotic leak (8.7%), incisional hernia (13%), fistula (8.7%), respiratory failure (8.7%), and perforation (4.4%). The median LOS following RYGB was six days compared to 1.5 ± 0.5 days following StomaphyX endoscopic revision. 

## 4. Discussion

This study retrospectively compares the use of two distinct treatment strategies in patients with failed VBG at a single institution. Formal conversion of the VBG to a RYGB in morbidly obese patients results in marked weight loss, however, with considerable morbidity. On the other hand, endoscopic revision with the StomaphyX device in morbidly obese patient produced less weight loss, with minimal morbidity. 

Similar to our findings, Gagné et al. reported that 38% of patients converted to RYGB had either early or late complications, including anastomotic leak, abscess, stricture, bleeding, and respiratory failure. Other studies have also suggested increased complication rates ranging from 12% to 41%, following revisional surgery in previously failed restrictive bariatric procedures [10, 11]. 

Endoscopic revision of failed VBG via StomaphyX device is a novel treatment strategy. Endoscopic treatment avoids the need for operative revision and related intra-abdominal complications, such as anastomotic leakage. Two common reasons for VBG failure are enlargement of the gastric pouch and staple line dehiscence [14]. The StomaphyX device is used to decrease the size of the gastric pouch by approximating and immobilizing two or more serosal surfaces through tissue fastening [13]. Mikami et al. reported successful use of the StomaphyX device in 39 patients to reduce the size of the gastric pouch following RYGB [13]. These authors reported a 10 kg weight loss at one-year followup with no major complications and no mortalities. Leitman et al. also reported a 7.3 kg weight loss at one-year followup and no major complications, following endoscopic revision with the StomaphyX device [15].

The two main limitations of our study are duration of followup and a small sample size. There are also other potentially viable options such as revision with an adjustable gastric band that were not included. With these major limitations its not possible to make any definitive recommendations about the use of StomaphyX. It does, however, present an interesting debate about the relative importance of less complications versus more weight loss. 

## 5. Conclusion

In conclusion, endoscopic revision via the StomaphyX device is a safe revisional treatment strategy in morbidly obese patients that have failed VBG. In addition, endoscopic revision may be a reasonable initial approach to failed VBG, with low complication rates. Further studies are needed to clarify the role of the StomaphyX pouch reduction in patients with failed VBG. 

## Figures and Tables

**Table 1:** Patient characteristics of failed VBG patients following StomaphyX or RYGB (*N* = 37).

Characteristic	StomaphyX (*N* = 14)	RYGB (*N* = 23)
Age (years)	46.4 (6.7)	49.0 (8.0)
Sex (% female)	93	78
Preoperative weight (kg)	119.5 (25.9)	140.3 (32.1)
Preoperative BMI (kg/m^2^)	43.4 (9.7)	49.8 (8.4)

RYGB: Roux-en-Y gastric bypass; Standard Deviation indicated in brackets.

**Table 2:** Complications following VBG revision by either StomaphyX or RYGB.

Type of complication	StomaphyX (*N* = 14)	RYGB (*N* = 23)
Death	0	1
Anastomotic leakage	N/A	2
Fistula	0	2
Wound infection/abscess	N/A	3
Cardiopulmonary failure	0	2
Minor complications	4*	6

Total	4	16

N/A: not applicable. *Short-term nausea and headache.
